# Astrocyte‐mediated regulation of multidrug resistance p‐glycoprotein in fetal and neonatal brain endothelial cells: age‐dependent effects

**DOI:** 10.14814/phy2.12853

**Published:** 2016-08-22

**Authors:** Stephanie Baello, Majid Iqbal, William Gibb, Stephen G. Matthews

**Affiliations:** ^1^ Department of PhysiologyFaculty of MedicineUniversity of TorontoTorontoOntarioCanada; ^2^ Department of Obstetrics and GynecologyFaculty of MedicineUniversity of OttawaOttawaOntarioCanada; ^3^ Department of Cellular and Molecular MedicineFaculty of MedicineUniversity of OttawaOttawaOntarioCanada; ^4^ Department of Obstetrics and GynecologyFaculty of MedicineUniversity of TorontoTorontoOntarioCanada; ^5^ Department of MedicineFaculty of MedicineUniversity of TorontoTorontoOntarioCanada

**Keywords:** Astrocytes, blood–brain barrier, brain endothelial cells, fetus, glial cells, P‐glycoprotein

## Abstract

Brain endothelial cells (BECs) form a major component of the blood–brain barrier (BBB). In late gestation, these cells express high levels of the multidrug transporter p‐glycoprotein (P‐gp; encoded by *Abcb1*), which prevents the passage of an array of endogenous factors and xenobiotics into the fetal brain. P‐gp levels in the BECs increase dramatically in late gestation, coincident with astrocyte differentiation. However, the role of astrocytes in modulating P‐gp in the developing BBB is unknown. We hypothesized that factors produced by astrocytes positively regulate P‐gp in BECs. Astrocytes and BECs were isolated from fetal and postnatal guinea pigs. Levels of *Abcb1* mRNA and P‐gp were increased in BECs co‐cultured with astrocytes compared to BECs in monoculture. Moreover, postnatal astrocytes enhanced P‐gp function in fetal BECs but fetal astrocytes had no effect on postnatal BECs. These effects were dependent on secreted proteins with a molecular weight in the range of 3–100 kDa. LC/MS‐MS revealed significant differences in proteins secreted by fetal and postnatal astrocytes. We propose that astrocytes are critical modulators of P‐gp at the developing BBB. As such, aberrations in astrocyte maturation, observed in neurodevelopmental disorders, will likely decrease P‐gp at the BBB. This would allow increased transfer of P‐gp endogenous and exogenous substrates into the brain, many of which have neurodevelopmental consequences.

## Introduction

The blood–brain barrier (BBB), present at the level of cerebral capillary endothelium, is an organ‐specific barrier that protects the brain. The BBB is formed by a specialized endothelium that displays barrier characteristics, able to regulate the movement of molecules from blood into brain compartments. Tight junctions and adheren junctions form between brain endothelial cells (BECs) and limit paracellular movement of proteins and large hydrophilic molecules across the BBB (Abbott et al. 2010). These tight junctions are present in the embryonic brain and have been shown to be functionally effective (Mollgoard and Saunders 1975; Bauer et al. 1993). Lipid‐soluble substances can pass the BBB unless actively removed by transporters located on the luminal surface of BECs. P‐glycoprotein (P‐gp) is the highest expressed transporter at the BBB (van Asperen et al. 1996; Kemper et al. 2003; von Moltke et al. 2004). P‐gp is encoded by a single gene, *Abcb1,* in humans and guinea pigs and two genes, *Abcb1a/Abcb1b*, in rats and mice (Pappas et al. 2014). Deficiency in P‐gp increases the passage of its substrates into the fetal brain, resulting in teratogenesis (Lankas et al. 1998). This is clinically relevant as approximately 60% of pregnant women take prescription drugs, many of which are substrates of P‐gp (Andrade et al. 2004, 2006; Daud et al. 2015). Additionally, P‐gp regulates the movement of endogenous compounds involved in brain development, such as cytokines and steroid hormones (Bain et al. 1997; Kim and Benet 2004).

Protection of the developing brain via P‐gp at the BBB becomes essential during the transition from fetal to neonatal life as the protection afforded by placental P‐gp is lost (Kalabis et al. 2005, 2009; Lye et al. 2013). Our group has shown, in mice and guinea pigs, that in early gestation, brain microvessels express very low amounts of P‐gp (Petropoulos et al. 2010; Iqbal et al. 2011). However, levels of P‐gp dramatically increase in late gestation and postnatal life. This upregulation in P‐gp is coincident with astrocyte differentiation, a process by which immature astrocytes proliferate and begin to extensively ensheathe the abluminal surface of brain microvessels (Dobbing and Sands 1979; Risau and Wolburg 1990; Byrnes et al. 2003). BECs interact very closely with astrocytes as astrocytic‐end feet ensheathe 99% of the abluminal surface of brain capillaries (Abbott 2002; Mathiisen et al. 2010).

Previous studies have shown that astrocytes are key regulators in the induction and maintenance of BBB characteristics of brain endothelium. BECs that have been co‐cultured with astrocytes show enhanced activity of BBB‐specific enzymes and transporters, and reduced paracellular transport compared to BECs cultured without astrocytes (Hayashi et al. 1997; Sobue et al. 1999; Abbott 2002; Berezowski et al. 2004; Haseloff et al. 2005). Moreover, astrocytes are capable of inducing these properties in endothelial cells derived from nonneural tissue (Janzer and Raff 1987; Hayashi et al. 1997; Kuchler‐Bopp et al. 1999). Astrocyte‐conditioned medium (ACM) can mimic these effects implicating astrocyte‐derived factors. However, many of these studies use cells derived from adult animals, or co‐culture cells from different ages and in some cases, from different species.

In this study, we investigated the effect of astrocytes on P‐gp function and expression at the developing BBB. Our laboratory has developed an in vitro BBB model comprised of BECs and astrocytes derived from fetal and postnatal guinea pigs. We utilized this model to investigate and characterize the astrocyte‐derived factors responsible for upregulating P‐gp at the developing BBB. We hypothesized that astrocytes would enhance P‐gp expression and function in BECs, but that the magnitude of this effect would vary with developmental age.

## Materials and Methods

### Animals

Twelve‐week‐old female Dunkin‐Hartley‐strain guinea pigs were purchased from Charles River Canada Inc. (St. Constant, Quebec, Canada) and were bred as described previously (Dean and Matthews 1999). 2‐week‐old male Dunkin‐Hartley‐strain guinea pigs were also purchased from Charles River Canada Inc. The guinea pig is an ideal animal model for investigating the interaction of astrocytes and BECs in regulating BBB characteristics. In contrast to the mouse, the guinea pig has a relatively long gestation period (~68 days) and has similar placentation to humans. Our laboratory has shown that unlike other rodents, P‐gp is encoded by a single gene, *Abcb1* in the guinea pig (Pappas et al. 2014). Moreover, guinea pig brain development follows a similar pattern to human brain development, such that the fetal brain growth spurt, due to rapid gliogenesis occurs in utero (Dobbing and Sands 1979; Byrnes et al. 2003). There are also many advantages to using guinea pigs for establishing an in vitro co‐culture model of the BBB. Guinea pigs give birth to large fetuses (~100 g at term) that provide sufficient brain tissue for in vitro analysis. Fetal brain tissue derived at mid‐gestation is sufficient to yield both primary BECs and astrocyte cultures. These characteristics have allowed us to generate a co‐culture model, using primary BECs and astrocytes derived from guinea pigs at various stages of fetal and neonatal development. Animal protocols used in the following studies were approved by the Animal Care Committee at the University of Toronto and in accordance with the Canadian Council on Animal Care.

### Isolation and culture of guinea pig primary brain endothelial cells

Isolation of BECs from gestational day (GD) 50 and postnatal day (PND) 14 guinea pigs was carried out as previously described (Iqbal et al. 2011). These time‐points in development were chosen as our group has found that P‐gp expression is low at GD50 and high at PND14 at the BBB (Iqbal et al. 2011). Briefly, guinea pigs were anesthetized with isoflurane (Baxter Corp Mississauga, Ontario, Canada). Once anesthetized, guinea pigs were decapitated and brain tissue was excised, meninges removed, and brain tissue halved. One half was used for BEC isolation, while the other half was used to isolate astrocytes as explained below. Brain tissue was minced and homogenized (Potter‐Elvehjem Tissue Grinder; Sigma, St. Louis, MO). The homogenate was suspended in dextran solution (17.5%; Sigma) and centrifuged (4000*g* for 15 min). Microvessel pellet was mixed with type I collagenase solution (1 mg/mL; Sigma) and digested for 30 min at 37°C. At the end of digestion, the solution was centrifuged (1000*g*, 10 min), the pellet was resuspended in Dulbecco's Modified Eagle Medium (DMEM; Wisent Inc., Saint‐Jean‐Baptiste, Quebec, Canada) supplemented with 20% fetal bovine serum (Wisent Inc.), plated on 0.5% gelatin‐coated 75 mm^2^ tissue culture flasks (Becton‐Dickinson BD Biosciences, Franklin Lakes, NJ) and grown at 37°C in 5% CO_2_/air. BECs were frozen in liquid nitrogen. BECs isolated and cultured in this way have been previously characterized (Iqbal et al. 2011). BECs derived from different stages of development retain relative developmental levels of P‐gp activity and levels of *abcb1* mRNA (Iqbal et al. 2012).

### Isolation and culture of guinea pig primary astrocytes

Astrocytes were extracted from GD50 and PND14 guinea pigs, using a modified protocol (Babu et al. 2011). Brain tissue was cut into small pieces and digested in papain (2.5 U/mL; Worthington Biochemical Corp., Lakewood, NJ) and DNAse (250 U/mL; Worthington Biochemical Corp.) at 37°C for 30 min. After incubation, papain/DNAse was removed by centrifugation (1000*g*, 10 min), the pellet was resuspended in Neurobasal A medium (Invitrogen, Carlsbad, CA) and loaded onto a column containing glass beads (5 mm; Thermo Fisher Scientific, San Jose, CA). The filtrate was plated on rat tail collagen‐coated 75 mm^2^ tissue culture flasks (BD Biosciences) and grown at 37°C in 5% CO_2_/air. Astrocytes were frozen in liquid nitrogen until use.

### Astrocyte characterization

Astrocytes from GD50 and PND14 guinea pigs were plated on 8‐well tissue culture slides (BD Biosciences). At 80% confluence, the medium was removed and cells were washed with HBSS (Invitrogen). Cells were fixed using paraformaldehyde (4%, Sigma; 22°C, 30 min), washed in Tris‐buffered saline (0.01 mol/L, pH7.5; Sigma) containing Triton X‐100 (0.1% TBS‐Triton; Sigma) and blocked in normal goat serum (NGS; 1.5% in TBS Triton; Invitrogen) for 2 h. Astrocytes were then incubated with primary antibody (diluted in NGS/TBS‐Triton) overnight at 4°C. Primary antibodies: rabbit anti‐von Willebrand Factor (vWF; 1:200; Sigma), mouse anti‐glial fibrillary acidic protein (GFAP; 1:100; Cell Signaling Technology, Inc., Danvers, MA), rabbit anti‐melanoma‐associated chondroitin sulfate proteoglycan (Ng2; 1:100; Cell Signaling Technology, Inc.), mouse anti‐Nestin (1:200; abcam, Cambridge, United Kingdom), rabbit anti‐aquaporin 4 (AQP4; 1:200; abcam), mouse anti‐synaptophysin (1:100; Millipore, Billerica, MA). Cells were washed and subsequently incubated with Alexa Fluor 488‐ or 555‐conjugated goat or secondary antibody (1:500 in NGS/TBS‐Triton; Invitrogen) depending upon the primary antibody species. As a negative control, cells were incubated with mouse IgG or rabbit IgG following secondary antibody. After the final washes, cells were mounted using mounting media with DAPI (Vector Laboratories, Burlingame, CA) and coverslipped.

### Guinea pig co‐culture and measurement of tight junction function

BECs and astrocytes were plated on 75 mm^2^ tissue culture flasks (BD Biosciences) in respective media described above and grown at 37°C in 5% CO_2_/air. To establish co‐cultures, using GD50 and PND14 BECs and astrocytes, astrocytes were plated in the basolateral compartment of Transwell plate (polyethylene terephthalate (PET); Corning Inc., Corning, NY) coated with rat‐tail collagen at a seeding density of 1 × 10^4^ cells/cm^2^. BECs were plated on the Transwell insert containing 0.45 *μ*m pores (PET; Corning) coated with 0.5% gelatin at a seeding density of 1 × 10^4^ cells/cm^2^. The insert membrane growth surface was 0.33 cm^2^. In co‐culture, cells were grown in BEC media. transendothelial electrical resistance (TEER) measurements were undertaken every 24 h using Millipore Millicell ERS probe MERSSTX01 and ERS‐2 Epithelial volt‐ohm meter (Millipore, Billerica, MA). Cells were washed twice with tyrode solution (Sigma). Chopstick electrodes were inserted into the donor and receiver chambers. Final resistance was calculated by subtracting the resistance of blank filters. Cell number between treatments was constant as measured by a trypan blue.

Tight junction function was also assessed, using a permeability assay with FITC‐dextran (70 kDa; Sigma), which was used to mimic serum albumin (~67 kDa). This assay was carried out after 72 h in culture to further corroborate TEER values. Cells were washed twice with tyrode solution (Sigma). FITC‐dextran was added to the apical compartment (0.01 mg/mL) and after 1 h, 100 *μ*L of medium was removed from the basal compartment and substituted with the same amount of tyrode solution (Sigma). Samples were collected on a 24‐well tissue culture plate (BD Biosciences), and fluorescence was measured at Ex/Em: 485/530 nm. Analyses using low molecular weight dextran were not carried out due to limited amount of cells.

### P‐gp functional assay

P‐gp activity was measured using calcein‐AM as described previously (Feng et al. 2008; Iqbal et al. 2011; Baello et al. 2014). BECs were washed twice with Tyrode solution (Sigma) incubated at 37°C. Co‐cultured BECs plated on inserts were transferred into new 24‐well tissue culture plates (BD Biosciences) and washed. Subsequently, BECs were incubated with calcein‐AM (1 *μ*mol/L calcein‐AM; Sigma) for 1 h at 37°C in 5% CO_2_/air. After incubation, BECs were placed on ice, washed twice with tyrode solution (Sigma), and lysed with ice‐cold 1% Triton X‐100 (Sigma) lysis buffer. Intracellular calcein was measured, using a spectrophotometer (Ex/Em: 485/510 nm). Mean background fluorescence was subtracted from all control and treated readings. Control values were divided by treatment values and expressed as fold‐change from control.

To validate that the effects of co‐culturing were specific to P‐gp, cells were incubated with verapamil. Verapamil is an L‐type calcium channel blocker that has been shown to be a competitive inhibitor of P‐gp (Weiss et al. 2003). Cells were washed with Tyrode and then subsequently incubated for 1 h with either calcein‐AM (1 *μ*mol/L) or calcein‐AM with 10^−4^ m verapamil (10 *μ*mol/L; Sigma). Cells were then washed, lysed, and calcein was measured, as described above.

### Quantification of mRNA expression

BECs were monocultured or co‐cultured on 24‐well Transwell inserts as described above (Corning). On Day 3 of co‐culture, BECs were trypsinized (0.05% trypsin‐EDTA [Invitrogen]) and centrifuged (1000*g*, 5 min). RNA was extracted, using TRIzol reagent (Invitrogen) as per the manufacturer's protocol. RNA was converted into cDNA via High Capacity cDNA Reverse Transcription kit (Applied Biosystems, Waltham, MA) as per the manufacturer's protocol using the C1000 Thermal Cycler. Thereafter the cDNA (100 ng) was mixed with primer probes (*Abcb1*: Forward – CAATCTGGGCAAAGATACTG, Reverse – CAAGTTCTTTGCTTTGTCCTC [Ensembl ID: ENSCPOT00000012540]; *β*‐actin: Forward – TTTACAATGAATTGCGTGTG, Reverse –ACATGATCTGGGTCATCTTC [Ensembl ID: ENSCPOT00000013600])) and SsoFast EvaGreen Supermix (Bio‐rad Laboratories, Inc., Hercules, CA). Samples were prepared in triplicate. Primer sets were designed, using Autoprime Primer Design (Gunnar Wrobel & Felix Kokocinski; Cambridge, United Kingdom) and synthesized by Integrated DNA Technologies (Coralville, IA). Samples were loaded onto CFX96 Real‐Time System (Bio‐Rad). Relative mRNA expression was calculated as the gene of interest expression normalized [ΔΔc(t)] to the reference gene expression, *β*‐actin. Expression levels of *β*‐actin did not change with age or co‐culturing conditions.

### Collection of astrocyte‐conditioned medium

Astrocytes from GD50 and PND14 guinea pigs were plated on 150 mm^2^ petri dishes at a seeding density of 1 × 10^4^ cells/cm^2^. Once confluent, astrocytes were washed twice with HBSS (Invitrogen) and media was changed to phenol‐red free Neurobasal A medium (Invitrogen). After 24 h, astrocyte conditioned media (ACM) was collected, centrifuged at 1000*g* for 10 min, and passed through a 0.22 *μ*mol/L filter. ACM was frozen at −80°C until use.

### Heat‐inactivation, protease treatment, and exosome‐depletion of astrocyte‐conditioned medium

ACM was subjected to heat inactivation at 100°C for 4 h. For protease treatment, ACM was incubated with trypsin (200 *μ*g/mL; Sigma) at 37°C for 1 h and subsequently incubated with trypsin inhibitor (400 *μ*g/mL; Sigma) at 37°C for 30 min. As a control, trypsin and trypsin inhibitor were mixed and incubated at 37°C for 1 h and 30 min and then added to ACM. To eliminate exosomes, ACM was centrifuged at 10,0000*g* for 6 h and the supernatant collected.

### Fractionation of astrocyte‐conditioned medium

To obtain fractions of ACM‐containing soluble factors of varying molecular weights, ACM was fractionated, using Centrifugal Filter Devices with different MWCO (Molecular Weight Cut‐Offs) Ultracel membranes (Millipore). First, 15 mL of ACM was loaded onto the centrifugal filter device with Ultracel membranes with a 100 kDa cut‐off and centrifuged at 2700*g* for 35 min. The retentate was collected and resuspended in the same volume of Neurobasal A medium (Invitrogen) as the loaded sample. The filtrate was collected and resuspended in the same volume of Neurobasal A medium as the loaded sample and subsequently loaded onto a column with a 30 kDa membrane cut‐off and centrifuged at 2700*g* for 35 min. The retentate and filtrate were again collected and resuspended in the same volume of medium as the loaded sample. The filtrate was loaded onto a column with a 3 kDa membrane cut‐off and centrifuged at 2700*g* for 35 min. BECs were then treated with these fractions of ACM containing proteins of varying molecular weights and P‐gp function was assessed.

### In‐solution digestion

Proteins in ACM were denatured, reduced, and alkylated prior to digestion and LC‐MS/MS. Briefly, protein (10 mg) was incubated with urea (8 mol/L, Sigma), 50 mmol/L Tris‐HCl (Sigma; pH 8), and 4 mmol/L DTT (Sigma) at 60°C for 30 min and then incubated with iodoacetamide (100 mmol/L; Sigma, 22°C, 15 min). The samples were subsequently diluted five times with 0.1 mol/L Tris–HCl pH 8.3 (Sigma) and digested, using trypsin (1:50 w/w; Sigma; 37°C, 16 h). Samples were desalted, using Pierce C18 Spin Tips (Thermo Fisher Scientific) as per the manufacturer's protocol.

### LC‐MS/MS analysis

Tryptic digests were analyzed on an Orbitrap analyzer (Q‐Exactive, Thermo Fisher Scientific) outfitted with a nanospray source and EASY‐nLC nano‐LC system (Thermo Fisher Scientific). Lyophilized peptide mixtures were dissolved in 0.1% formic acid and loaded onto a 75 *μ*m × 50 cm PepMax RSLC EASY‐Spray column filled with 2 *μ*mol/L C18 beads (Thermo Fisher Scientific) at a pressure of 800 Bar. Peptides were eluted over 60 min at a rate of 250 nL/min, using a 0–35% acetonitrile gradient in 0.1% formic acid. Peptides were introduced by nano‐electrospray into the Q‐Exactive mass spectrometer (Thermo Fisher Scientific). The instrument method consisted of one MS full scan (400–1500 *m*/*z*) in the Orbitrap mass analyzer with an automatic gain control (AGC) target of 1E6, maximum ion injection time of 120 msec and a resolution of 70,000 followed by 10 data‐dependent MS/MS scans with a resolution of 17,500, an AGC target of 1E6, maximum ion time of 120 msec, and one microscan. The intensity threshold to trigger an MS/MS scan was set to 1.7E4. Fragmentation occurred in the HCD trap with normalized collision energy set to 27. The dynamic exclusion was applied, using a setting of 10 sec.

### Protein Identification

Raw data generated by LC‐MS/MS (peaklists generated by Xcalibur 2.2) were analyzed, using Xcalibur (Thermo Fisher Scientific) for ion current analysis, and were searched against the guinea pig SwissProt UniProt protein‐database (containing 20392 entries). Database searching was undertaken using SEQUEST version 1.4.0.288 (through Proteome Discoverer, Thermo Fisher Scientific). The data was then imported into Scaffold 4.3.4 (Proteome Software, Portland, OR) and X!Tandem CYCLONE (2010.12.01.1) database searching was performed. These databases were searched with a parent ion tolerance of 10.0 PPM, and a fragment mass tolerance of 0.6 Da. Peptide identifications were accepted if they could be established at greater than 95% probability by the Scaffold Local FDR algorithm. Protein identifications were accepted if they could be established at <95% probability and contained at least three identified peptides. Protein probabilities were assigned by the ProteinProphet algorithm (Nesvizhskii et al. 2003).

### Protein Quantification

Proteins were quantified based on the average MS signal response for the three most intense tryptic peptides for each protein and expressed as percentage of total protein. Briefly, the MS files were searched as listed above and the SEQUEST result files were used to generate a spectral library in Skyline 2.1.0.4936 (MacLean et al. 2010), using the guinea pig sequences from SwissProt as background proteome. Employing the same parameters as for the SEQUEST search, the three most abundant peptides for each protein were manually chosen from all peptides available in the spectral library. After data import, the chromatographic traces (extracted ion chromatograms) were manually inspected and adjusted where needed to correct wrongfully assigned peaks. Proteins identified with less than two peptides were not included in the quantification. The relative abundance of proteins quantified in three samples was calculated as the average MS intensity for the two peptides for each protein divided by the sum of the average signal for all quantified proteins in the sample. Relative abundance of protein was displayed as fold change from protein levels found in GD50 ACM.

### Statistics

Statistical analyses were performed using Prism (GraphPad Software, Inc., San Diego, CA). Effect of co‐culture or ACM on tight junction function and *Abcb1*/P‐gp was analyzed, using Student's unpaired *t*‐test. Effect of heat‐inactivated and exosome‐depleted ACM was also analyzed via Student's unpaired *t*‐test. Effect of age‐match and different‐aged co‐culture, protease‐treated ACM, and fractionation of ACM data was analyzed, using one‐way ANOVA, followed by Newman–Keuls post hoc analyses. Significance was set at *P *<* *0.05. Each treatment group consisted of cells derived from 5‐8 animals.

LC‐MS/MS data was analyzed, using MSstats (3.1.4; Vitek Lab, Purdue University). Three biological replicates of ACM were collected at each gestational age. Proteomic differences between GD50 and PND14 ACM were evaluated for statistical significance (*P < *0.05) by Student unpaired *t*‐tests, and corrected for multiple testing, using the Benjamini–Hochberg correction.

## Results

### Characterization of fetal and postnatal astrocytes

**Figure 1:**
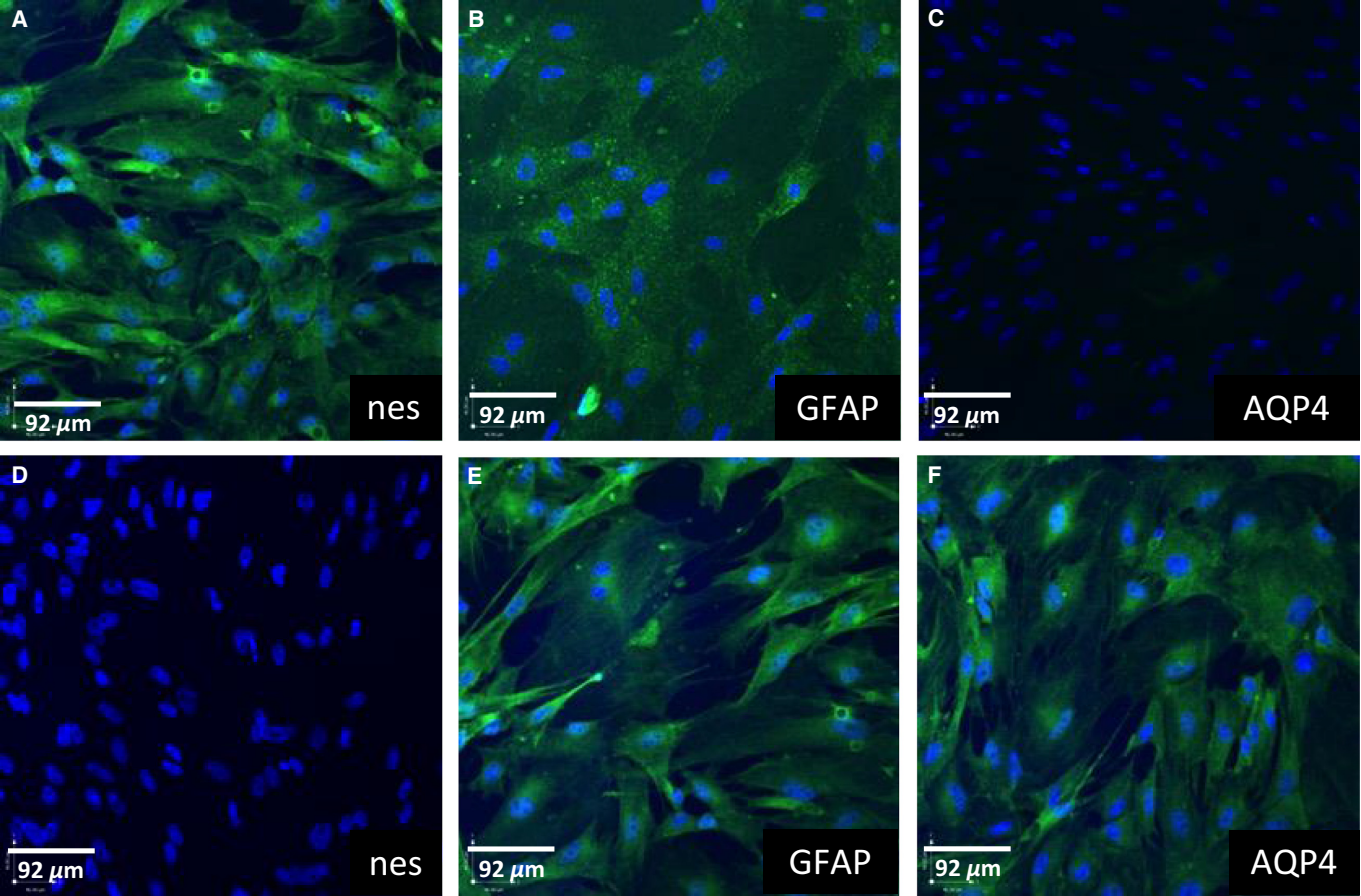
Fluorescent immunocytochemical images of astrocytes derived from gestational day (GD) 50 (A–C) and postnatal day (PND) 14 (D–F) male guinea pigs. Cells were stained for Nestin (nes), glial fibrillary acidic protein, and aquaporin‐4 (AQP4). Cell nuclei were stained by 4′,6‐diamidino‐2‐phenylindole (blue).

Astrocytes were characterized via immunofluorescence for markers of immature (Nestin) and mature astrocytes (GFAP [glial fibrillary acidic protein] and AQP4). Astrocytes derived from GD50 guinea pigs expressed Nestin and GFAP (>95%), but stained negatively for AQP4 (Fig. 1A–C). In contrast, PND14 astrocytes were positive for GFAP and AQP4 (>95%) but did not express Nestin (Fig. 1D–F). Both GD50 and PND14 astrocytes did not express (<1%) for synaptophysin (neuron marker), vWF **(**BEC marker), or Ng2 (pericyte marker)(data not shown), indicating little contamination by other CNS cell types.

### Effect of astrocytes on tight junction function in BECs

**Figure 2:**
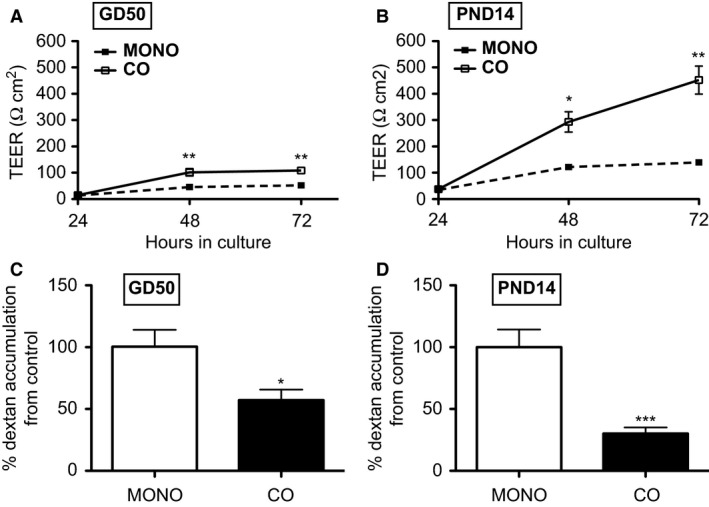
Tight junction function in monocultured (MONO) or co‐cultured (CO) BECs derived from gestational day (GD) 50 and postnatal day (PND) 14 guinea pigs (*n* = 8/group). Tight junction function was assessed using TEER after 24 h, 48 h, and 72 h in culture (A, B). Data from TEER experiments is displayed as mean ± SEM. After 72 h in culture, FITC‐dextran was also used to measure tight junction function (C, D). Data is expressed as % dextran accumulation from control (monocultured BECs). A significant difference from monocultured cells is indicated by *P <* *0.05; **P <* *0.01; ***P <* *0.001. BECs, Brain endothelial cells; TEER, transendothelial electrical resistance.

Co‐culture of age‐matched cells increased TEER in GD50 (*P < *0.01) and PND14 (*P < *0.05) BECs compared to monocultured cells after 48 h and 72 h (Fig. 2A and B). After 72 h in culture, co‐cultured PND14 BECs attained a TEER of approximately 452 Ω cm^2^, while co‐cultured GD50 BECs displayed a TEER of 109 Ω cm^2^ (Fig. 2A and B). These results were corroborated with FITC‐dextran permeability assay. Co‐cultured GD50 and PND14 BECs also demonstrated decreased permeability to FITC‐dextran (70 kDa) after 72 h in culture compared to monocultured BECs, approximately 60% and 30%, respectively (*P *< 0.05, *P < *0.001) (Fig. 2C and D).

### Effect of astrocytes on P‐gp activity and levels of Abcb1 mRNA in BECs

**Figure 3:**
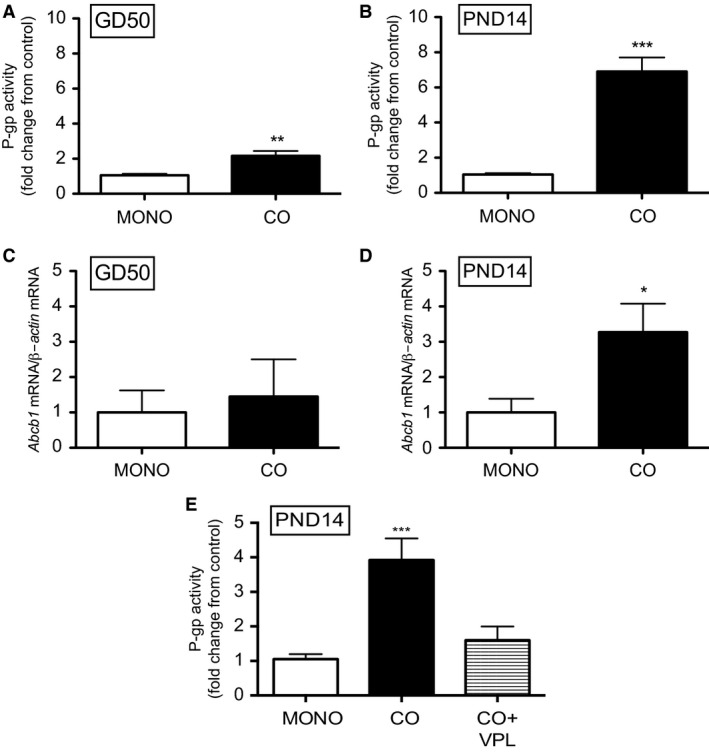
P‐gp activity (A, B) and levels of *Abcb1* mRNA (C, D) in monocultured or co‐cultured BECs derived at gestational day (GD) 50 and postnatal day (PND) 14 (*n* = 8/group). P‐gp activity was also measured in co‐cultured BECs (E) that was exposed to P‐gp inhibitor, verapamil (VPL) (*n* = 5/group). P‐gp activity is displayed as fold change from control (monocultured BECs). *Abcb1* mRNA expression was normalized to *β‐Actin* and shown as fold change from monocultured BECs. Data are displayed as mean ± SEM. A significant difference from monocultured cells is indicated by *P < 0.05; **P <* *0.01; ***P < 0.001.

Since tight junction function was maximal after 72 h in culture, P‐gp activity and levels of *Abcb1* mRNA were measured in monocultured and co‐cultured BECs at this time‐point. P‐gp activity in GD50 and PND14 BECs co‐cultured with age‐matched astrocytes increased by approximately twofold (*P < *0.01) and sevenfold (*P < *0.001), respectively, compared to monocultured BECs (Fig. 3A and B). This corresponded to a threefold increase in *Abcb1* mRNA (*P < *0.05) in co‐cultured PND14 BECs compared to monocultured PND14 BECs (Fig. 3D). However, there was no change in *Abcb1* mRNA levels in co‐cultured GD50 BECs compared to monocultured GD50 BECs (Fig. 3C).

In order to demonstrate that this effect of co‐culturing was indeed specific to P‐gp, co‐cultured BECs derived from PND14 guinea pigs were incubated with calcein‐AM in the presence of P‐gp inhibitor, verapamil (VPL). This treatment attenuated the effects of co‐culturing on P‐gp function (Fig. 3E).

### Effect of age of astrocytes on P‐gp activity and levels of Abcb1 mRNA in BECs

**Figure 4:**
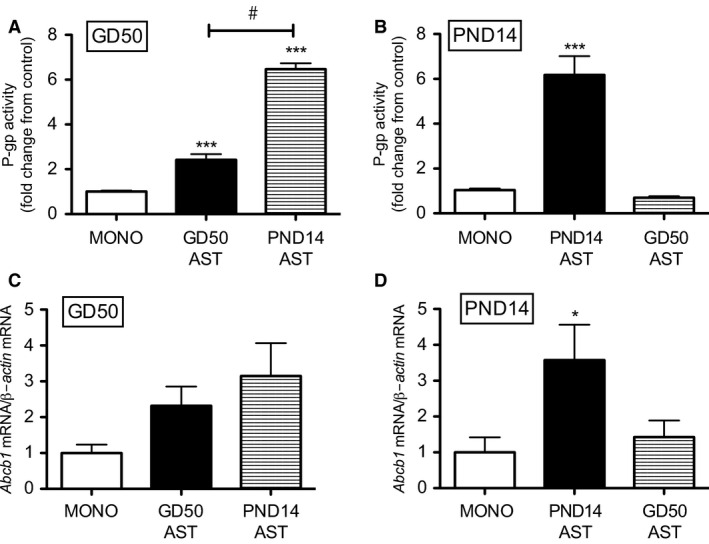
P‐gp activity (A, B) and levels of *Abcb1* mRNA (C, D) measured in gestational day (GD) 50 and postnatal day (PND) 14 BECs after monoculture, co‐culture with age‐matched astrocytes or co‐culture with different‐aged astrocytes (*n* = 8/group). P‐gp activity is displayed as fold change from control (monocultured BECs). *Abcb1* mRNA expression was normalized to *β‐Actin* and shown as fold change from monocultured BECs. Data are displayed as mean ± SEM. A significant difference from monocultured BECs is indicated by *P <* *0.05; ***P < 0.001. A significant difference between two co‐culture treatments is indicated by #*P < *0.01. BECs, Brain endothelial cells.

Co‐culture induced more dramatic changes in P‐gp activity and levels of *Abcb1* mRNA in PND14 cultures compared to GD50 cultures. Therefore, GD50 BECs were co‐cultured with PND14 astrocytes and vice versa. Co‐culturing GD50 BECs with PND14 astrocytes induced a greater increase in P‐gp function compared to co‐culturing with GD50 astrocytes (*P < *0.01; Fig. 4A). However, despite the enhancement in P‐gp activity in GD50 BECs after co‐culturing with PND14 astrocytes, there was no increase in levels of *Abcb1* mRNA compared to GD50 BECs that were monocultured or co‐cultured with GD50 astrocytes (Fig. 4C). Moreover, co‐culturing PND14 BECs with GD50 astrocytes did not increase in P‐gp function or levels of *Abcb1* mRNA in PND14 BECs (Fig. 4B and D).

### Effect of astrocyte‐secreted factors on P‐gp function in BECs

**Figure 5:**
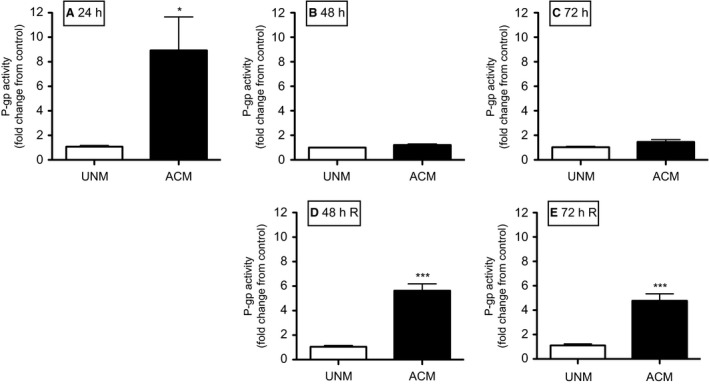
P‐gp activity in postnatal day (PND) 14 BECs exposed to unconditioned (UCM) or ACM for 24 h (A), 48 h (B) and 72 h (C). P‐gp activity was also measured in PND14 BECs to UCM or ACM, with the media being changed every 24 h (D, E). P‐gp activity is displayed as fold change from control (monocultured BECs) (*n* = 8/group). Data are displayed as mean ± SEM. A significant difference from control is indicated by *P < 0.05; ***P <* *0.001. ACM, Astrocyte‐conditioned medium; BECs, Brain endothelial cells.

To determine if the effects of co‐culture on P‐gp function were due to astrocyte‐derived factors, we exposed PND14 BECs to ACM from PND14 astrocytes. P‐gp activity was increased in PND14 BECs after 24 h of exposure compared to control BECs exposed to unconditioned medium (UNM) (*P < *0.05; Fig 5A). Surprisingly, the effects of ACM on P‐gp activity were not observed at the 48 h or 72 h time‐points (Fig. 5B and C). However, when ACM was changed every 24 h, the effects were rescued at the 48 h and 72 h time‐points (*P < *0.001*, P < *0.001; Fig. 5D and E).

**Figure 6:**
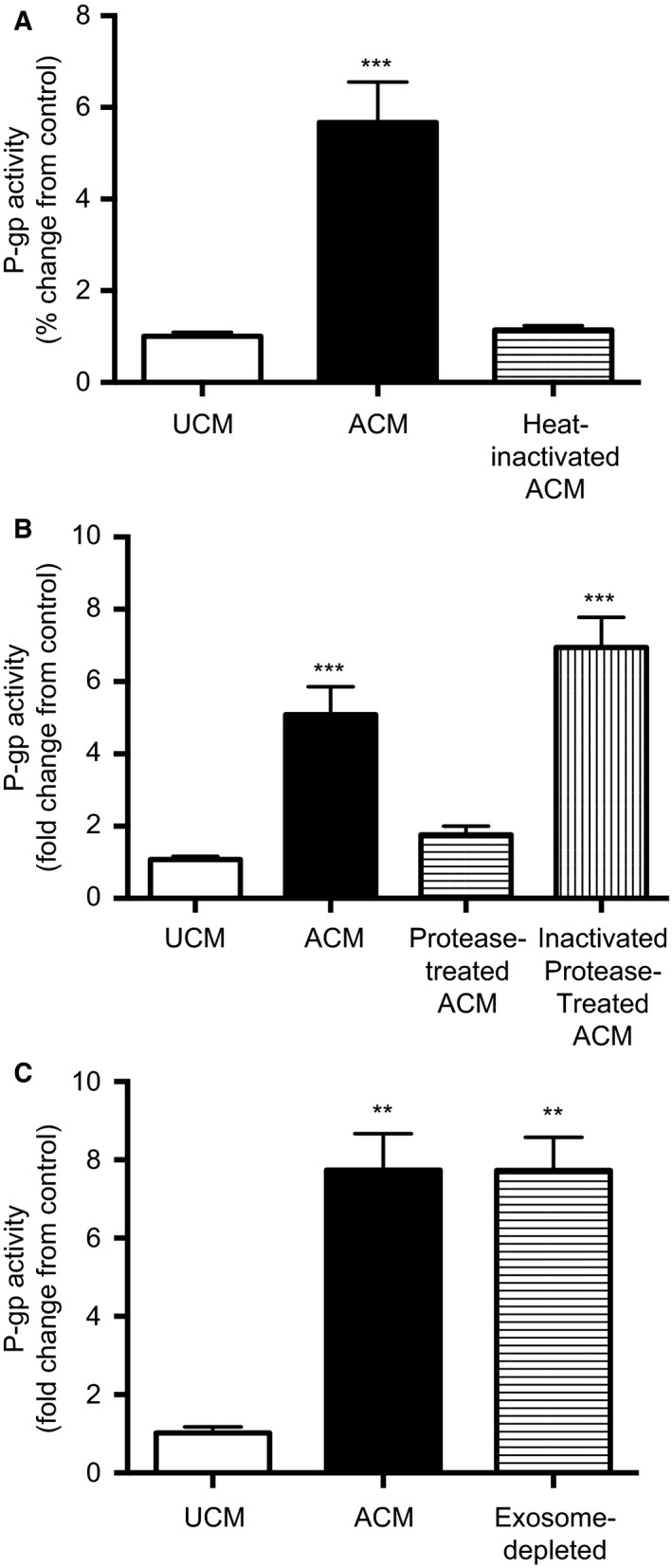
P‐gp activity in postnatal day (PND) 14 BECs exposed to heat‐inactivated (A), protease‐treated (B), or exosome‐depleted (C) ACM. ACM was subjected to heat‐inactivation at 100°C for 4 h. For protease treatment, ACM was incubated with trypsin (200 *μ*g/mL; Sigma) at 37°C for 1 h and subsequently incubated with trypsin inhibitor (400 *μ*g/mL; Sigma) at 37°C for 30 min. As a control, trypsin and trypsin inhibitor were mixed and incubated at 37°C for 1 h and 30 min and then added to ACM. To eliminate exosomes, ACM was centrifuged at 100,000*g* for 6 h and the supernatant collected. P‐gp activity is displayed as fold change from control (BECs exposed to unconditioned medium [UCM]) (*n* = 8/group). Data are displayed as mean ± SEM. A significant difference from control is indicated by **P <* *0.01; ***P <* *0.001. ACM, astrocyte‐conditioned medium; BECs, Brain endothelial cells.

To investigate whether the astrocyte‐secreted factors responsible for increasing P‐gp activity were proteins, ACM was subjected to heat‐inactivation and trypsin (protease) treatment. Heat‐inactivated ACM did not increase P‐gp activity in PND14 BECs after 24 h of exposure compared to BECs exposed to UNM (Fig. 6A). Treatment of ACM with trypsin also prevented the increase in P‐gp activity and the stimulatory‐effect was restored by inhibiting the protease (*P < *0.001; Fig. 6B). We also determined the potential role of exosome‐mediated communication between astrocytes and BECs (Fig. 6C). Exosome‐depletion of ACM did not affect its stimulatory affect on P‐gp activity (*P < *0.001) indicating that exosome protein cargo is not responsible for the stimulatory effect.

### Effect of ACM fractions on P‐gp function in BECs

**Figure 7:**
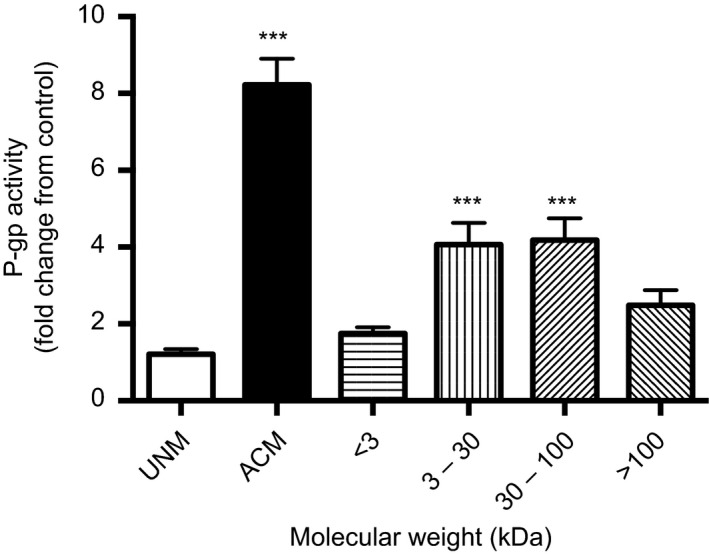
P‐gp activity in postnatal day (PND) 14 BECs exposed ACM containing soluble factors of varying molecular weights. ACM was fractionated using centrifugal filter devices with different MWCO (molecular weight cut‐offs) ultracel membranes. P‐gp activity is displayed as fold change from control (monocultured BECs) (*n* = 8/group). Data are displayed as mean ± SEM. A significant difference from control is indicated by ***P <* *0.001. ACM, astrocyte‐conditioned medium; BECs, Brain endothelial cells.

To determine the approximate size of the protein(s) responsible for upregulating P‐gp function in BECs, ACM was fractionated using successive filtration (molecular weight ranges: <3 kDa, 3–30 kDa, 30–100 kDa, >100 kDa). P‐gp activity was increased in PND14 BECs that were exposed to ACM containing 3–30 kDa and 30–100 kDa proteins, by approximately 4‐fold (*P < *0.001, *P < *0.001; Fig. 7) but was not significantly altered by ACM containing <3 kDa or >100 kDa protein fractions.

### Differential expression of proteins in GD50 and PND14 ACM

Using LC‐MS/MS, 467 proteins were identified in conditioned medium from GD50 and PND14 astrocytes. Of these proteins, 85 were found to be differentially secreted by PND14 and GD50 astrocytes, all of which were elevated in PND14 ACM compared to GD50 ACM (*P < *0.05; Table 1). The five principal proteins were Y‐box‐binding protein‐1 (YB‐1), high‐density lipoprotein‐binding protein, melanoma differentiation‐associated gene 20, SERPINE1 MRNA‐binding Protein 1 and Actin, Beta‐Like 2.

**Table 1:** List of proteins found to be upregulated in PND14 astrocyte‐conditioned medium (ACM) compared to GD50 (ACM) (*n* = 3/group)

Protein	Molecular weight (kDa)	Fold change
Y box‐binding protein 1	36	18.75
High‐density lipoprotein‐binding protein	141	16.92
Melanoma differentiation‐associated gene 20	18	14.09
SERPINE1 MRNA‐binding protein 1	45	8.51
Actin, beta‐like 2	42	6.26
Enolase 2 (Gamma, Neuronal)	47	6.17
KH domain containing, RNA‐binding, signal transduction associated 1	48	6.1
C‐Myc‐binding protein	40	5.79
Tropomyosin 2	33	5.78
Calsyntenin 2	107	5.5
Fructose‐bisphosphate aldolase	39	5.14
Reticulocalbin 3	37	5.04
MARCKS‐Like1	20	5.02
Profilin	15	4.75
Heat Shock 27 kDa Protein 1	23	4.56
Galectin	15	4.49
Von hippel–lindau‐binding protein 1	23	4.45
Neuropilin 2	105	4.18
Microtubule‐associated protein 1B	271	4.11
Tropomyosin alpha‐4 chain	50	4
Nestin	177	3.99
radixin	69	3.96
GTPase activating protein (SH3 Domain)‐binding protein 1	52	3.89
Transketolase	68	3.86
Small nuclear ribonucleoprotein 70 kDa (U1	52	3.86
Calponin	34	3.85
ATPase inhibitory factor 1	12	3.72
Heat shock 10 kDa protein 1	11	3.7
14‐3‐3 protein beta/alpha	28	3.65
Myosin, light chain 4	22	3.46
Lipoma‐preferred partner	66	3.46
Ependymin related 1	25	3.46
Phosphoribosylaminoimidazole carboxylase	47	3.41
Dihydropyrimidinase‐like 3	62	3.37
Actin‐related protein 2	45	3.31
Proteasome subunit alpha type	26	3.21
Eukaryotic translation elongation factor 1 Beta 2	25	3.18
Thioredoxin reductase 1	71	3.16
Septin 11	49	3.12
Septin 2	41	3.04
Ribosomal protein L30	13	3
A kinase (PRKA) anchor protein 12	191	2.98
Splicing factor proline/glutamine‐rich	76	2.91
Proteasome subunit alpha type	26	2.88
Alanyl‐TRNA synthetase	107	2.84
Peroxiredoxin 4	31	2.83
Fascin actin‐bundling protein 1	55	2.71
PDZ And LIM domain 7 (Enigma)	50	2.69
14‐3‐3 protein gamma	28	2.68
Protein disulfide‐isomerase	57	2.67
PDZ and LIM domain 5	64	2.61
Talin‐1	270	2.61
Proteasome subunit beta type	25	2.6
Endoplasmic Reticulum Protein 29	29	2.6
Triosephosphate isomerase	31	2.6
Fibromodulin	43	2.59
Dachsous cadherin‐related 1	346	2.55
Calcium‐activated nucleotidase 1	45	2.54
Filamin A, alpha	281	2.53
Binding immunoglobulin protein	72	2.52
Serine/threonine kinase receptor associated protein	38	2.5
Phospholipase B domain containing 2	65	2.5
Immunoglobulin superfamily containing leucine‐rich Repeat	46	2.49
Septin 7	51	2.45
Collagen, type I, alpha 2	129	2.44
Zyxin	61	2.43
Protein disulfide‐isomerase	57	2.4
Tropomodulin‐3	40	2.39
Myosin, heavy chain 9, nonmuscle	227	2.37
Collagen triple helix repeat containing 1	26	2.36
NAD(P)H dehydrogenase [quinone] 1	31	2.34
Adenosine deaminase	41	2.25
SH3 domain‐binding glutamic acid‐rich‐like protein	128	2.24
Proteasome subunit beta type	29	2.22
Lipopolysaccharide‐associated protein 1	71	2.2
Capping protein (Actin Filament), gelsolin‐like	38	2.18
Guanine nucleotide‐binding protein (G protein), beta polypeptide 2‐like, Proliferation‐inducing gene 21	35	2.17
Nucleoside diphosphate kinase	17	2.16
14‐3‐3 protein zeta/delta	28	2.15
Proteasome subunit alpha type	30	2.08
Microtubule‐associated protein RP/EB family member 1	30	1.95
Cartilage acidic protein 1	71	1.9
Malate dehydrogenase	36	1.87
Chaperonin‐containing tcp1, subunit 8 (Theta)	60	1.83
UDP‐glucose pyrophosphorylase 2	57	1.78

**Figure 8:**
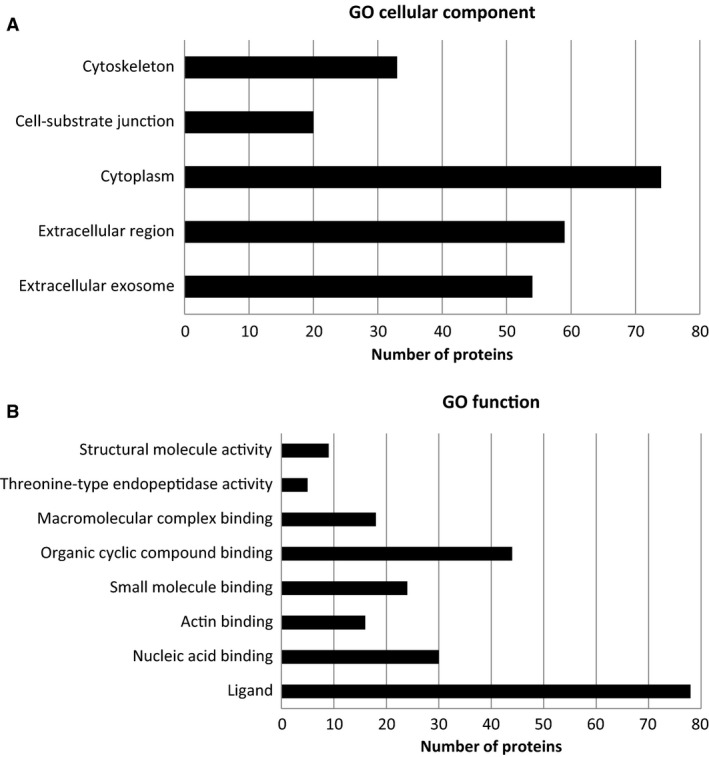
Bioinformatic analysis of differentially secreted proteins. Proteins were clustered based on gene ontology (GO) cellular component (A) and GO Function (B), using GO Term Finder. GO terms with *P < *0.01 are listed in the figure.

We further analyzed the differentially secreted proteins, using gene ontology (GO) Term Finder based on cellular component (Fig. 8A). Of these 85 proteins, 59 were identified as being extracellularly located and 54 were associated with extracellular exosomes. GO analysis also associated 74 proteins with cytoplasmic localization, with some proteins related to cytoskeletal and cell junction‐related proteins. Proteins were also analyzed based on GO function (Fig. 8B). Most proteins (~92%) were classified as ligand proteins, capable of binding to other molecules, such as small molecules (i.e., monosaccharaides), organic cyclic compounds, actin, nucleic acids, and macromolecules.

## Discussion

This is the first study to establish a co‐culture model, using BECs and astrocytes from two distinct developmental ages. Using this co‐culture model, we demonstrated that astrocytes enhance tight junction and P‐gp function in BECs. Moreover, postnatal astrocytes can dramatically upregulate P‐gp function in fetal BECs, while fetal astrocytes had no effect on P‐gp activity in postnatal BECs. Thus, the BBB‐inducing properties of astrocytes are dependent on developmental stage at which astrocytes were derived. We also found that ACM could mimic these effects on P‐gp function, suggesting a mechanism that involves soluble astrocyte‐derived factors. Using heat inactivation and protease treatment, we demonstrated that these astrocyte‐derived factors are dependent upon proteins. Fractionation of ACM indicated that these factors are in the molecular weight range of 3–100 kDa. Moreover, LC/MS‐MS identified 85 proteins that were significantly upregulated in PND14 ACM compared to GD50 ACM.

We have previously shown that isolated BECs retain their developmental characteristics (Iqbal et al. 2011). In the present study, we demonstrate that astrocytes also maintain properties appropriate to the age at which they were derived. Astrocytes derived at GD50 and PND14 express astrocyte markers appropriate to their developmental age. Nestin is expressed by GD50 astrocytes but not PND14 astrocytes. This is consistent with astrocyte development in vivo as Nestin is expressed by immature, developing CNS cells (Laywell et al. 2000; Lim et al. 2007). Nestin expression is downregulated and is transiently co‐expressed with GFAP during astrocyte differentiation (Sergent‐Tanguy et al. 2006). Astrocytes derived at PND14 but not GD50 express AQP4. AQP4 is the major water channel expressed in brain perivascular astrocyte processes and its expression is upregulated in vivo as differentiating astrocytic endfeet that make contact with the brain microvasculature (Nicchia et al. 2004). GFAP was expressed in both GD50 and PND14 astrocytes. GFAP may be important in astrocyte‐mediated upregulation of P‐gp expression and function at the BBB since GFAP knockout (KO) mice exhibit a structurally and functionally impaired BBB (Liedtke et al. 1996).

The majority of studies that have investigated the effects of astrocytes on the BBB have utilized co‐culture models composed of BECs and astrocytes from different ages or cells from different animal species. These studies demonstrated that astrocytes enhanced many BBB characteristics in co‐cultured BECs compared to monocultured BECs (Hayashi et al. 1997; Berezowski et al. 2004; Cecchelli et al. 2014). However, our study highlights the importance of the maturational status of astrocytes in the regulation of P‐gp developing BBB. We utilized a novel co‐culture model using BECs and astrocytes derived from the same animal at two precise time‐points in development to show that postnatal and fetal astrocytes can enhance P‐gp function in age‐matched BECs. However, postnatal astrocytes increased P‐gp function in fetal BECs but fetal astrocytes had no effect on postnatal BECs. Other studies have shown that the maturational state of astrocytes also affects neuronal development. Astrocytes derived from newborn and adult rats were shown to differ in their capacity to sustain neurite outgrowth in retinal ganglion cells in co‐culture (Smith et al. 1990; Bahr et al. 1995). Thus, the maturational status of astrocytes appears to influence both neuronal and BBB development.

A novel finding of our study was that PND14 astrocytes exhibit different effects on P‐gp function and levels of *Abcb1* mRNA in GD50 and PND14 BECs. There was strong correlation between *Abcb1* mRNA levels and P‐gp function in PND14 co‐cultures. However, in GD50 BECs, enhancement in P‐gp activity in GD50 BECs as a result of co‐culturing with age‐matched or different‐aged astrocytes, did not correspond to significant increases in *Abcb1* mRNA levels. One explanation for this observation may be that high variability in the data may have prevented significant differences to be statistically detected between monocultured and co‐cultured GD50 BECs. Alternatively, this data may indicate that there may be different mechanisms involved in upregulation of P‐gp function in GD50 and PND14 BECs. It is well‐established that P‐gp function can be enhanced without changes at the level of mRNA. This includes post‐translational modifications to P‐gp protein, such as glycosylation and phosphorylation, both of which lead to an increase in P‐gp function (Chambers et al. 1994; Germann et al. 1996; Draheim et al. 2010). P‐gp function can also be increased by the recruitment of intracellular storage of P‐gp in vesicles to the cell membrane (Fu and Arias 2012). Additionally, the epigenetic status of the *Abcb1* promoter may vary between GD50 and PND14 BECs. The density of methylation in the *Abcb1* promoter is negatively correlated with *Abcb1* expression in cancer cells (Fryxell et al. 1999; Baker and El‐Osta 2010). Our laboratory has shown that global DNA methylation in brain microvessels significantly decreases from GD50 to PND14 (M. Iqbal, J. Pappas and S.G. Matthews, unpubl. obs.). However, it has yet to be determined if these global changes in methylation include specific changes in promoter methylation of the *Abcb1* gene. Early in gestation, increased methylation of the *Abcb1* promoter may prevent binding of transcription factors, activated by astrocytes‐derived molecules, from increasing *Abcb1* mRNA levels in GD50 BECs. Moreover, GD50 and PND14 BECs may respond differently to astrocyte‐derived signals from PND14 astrocytes simply because they express different types or levels of receptors.

Utilizing ACM protease treatment and heat inactivation, we demonstrated that the astrocyte‐derived factor(s) is indeed a protein or dependent on a protein. We also showed that 3–30 kDa and 30–100 kDa PND14 ACM fractions can upregulate P‐gp function in PND14 BECs. These data indicate that more than one factor is likely responsible for this effect. Moreover, of the 85 proteins that were found to be significantly upregulated in PND14 ACM compared to GD50 ACM, 63 proteins fall into these molecular weight ranges. This suggests that astrocytes may regulate P‐gp function and *Abcb1* mRNA levels via multiple signaling pathways, which are activated by various astrocyte‐secreted proteins. Consistent with this hypothesis, studies have implicated many astrocyte‐derived factors in regulation of P‐gp at the adult BBB. These pathways include those linked to sonic hedgehog (shh), canonical Wnt/*β* ‐catenin, retinoic acid, and the transforming growth factor‐*β* (TGF‐*β*) superfamily (Prat et al. 2001; Abbott 2002; Dohgu et al. 2004; Liebner et al. 2008; Alvarez et al. 2011; Mizee et al. 2013; Baello et al. 2014). In silico pathway analysis revealed that a number of the proteins that we identified participate in these signaling pathways. However, our studies do not conclusively rule out the role of lipid or fatty acid derivatives in regulating P‐gp. For example, the stability of retinol, from which retinoic acid is derived, is dependent on proteins (Klaassen et al. 1999; N'Soukpoe‐Kossi et al. 2007) and retinol‐binding protein was identified in conditioned media from GD50 and PND14 astrocytes. Moreover, GO analysis classified most of the significantly upregulated proteins in PND14 ACM (Table 1) as ligand proteins, capable of binding to small molecules, nucleic acids, macromolecules and organic cyclic compounds. Further studies are needed to elucidate the identity of the factor(s) involved in astrocyte‐mediated upregulation in P‐gp function and expression at the fetal and postnatal BBB.

The most differentially expressed protein between GD50 and PND14 astrocytes was Y‐box‐binding protein (YB‐1), which was secreted approximately 18‐fold more by PND14 astrocytes compared to GD50 astrocytes. YB‐1 is a transcription factor that regulates genes such as *Abcb1* (Shen et al. 2011 *;* Dolfini and Mantovani 2013). However, recent studies indicate that many cell types release YB‐1 into the extracellular space (Frye et al. 2009; Raffetseder et al. 2011). Nanomolar concentrations were shown to stimulate proliferation and migration of rat mesangial cells and human kidney cells (Frye et al. 2009). This effect was mediated by YB‐1 interaction with EGF‐repeats of the Notch3 receptor, and activation of canonical Notch signaling (Rauen et al. 2009; D'Souza et al. 2010). Currently, very little is known about how Notch signaling regulates P‐gp function and expression. The only evidence of such regulation stems from cancer cells, in which Notch signaling is generally overactivated and is associated with increased P‐gp function (Capaccione and Pine 2013; Wu et al. 2014). For example, knockdown of Notch1 receptor leads to decreased *ABCB1* and *ABCC1* mRNA in cultured intrahepatic cholangiocarcinoma cells (Wu et al. 2014). Moreover, overexpression of Notch1 resulted in an increase in *ABCB1* mRNA in gastric human cancer cell lines (Hang et al. 2015). Future studies are needed to investigate the role of YB‐1 and Notch signaling in regulating P‐gp in normal tissues. Our laboratory was unable to carry out these experiments as there is no commercially available recombinant YB‐1 protein.

High‐density lipoprotein‐binding protein (HDLBP) was also found to be increased by approximately 16‐fold in PND14 ACM compared to GD50 ACM. HDLBP binds high‐density lipoprotein (HDL), which transports cholesterol, phospholipids, and triglycerides. A positive association between cellular cholesterol content and P‐gp function has been demonstrated in various cancer cell lines (Troost et al. 2004; Belli et al. 2009). However, the mechanism by which this occurs is unclear. HDLBP may mediate this effect as it is a secreted protein that can become anchored to the cell membrane through glycosylphosphatidylinositol‐enriched domains (Beigneux et al. 2007). Binding of cholesterol to HDLBP via HDL may elicit various signaling pathways (Fidge 1999). Consistent with this hypothesis, brain cholesterol content is maximal in late gestation and postnatal life, which is coincident with the surge in P‐gp at the BBB (Dietschy and Turley 2004). Additionally, HDLBP can bind to RNA and prevent its degradation (Fidge 1999). PND14 astrocytes may secrete more HDLBP, resulting in the stabilization of *Abcb1* mRNA in BECs. The link between cholesterol and P‐gp warrants further investigation, especially in the context of the developing BBB.

We also found that ACM has short‐term effects on P‐gp function in BECs, as these effects disappear at 48 h and 72 h. However, when ACM was replaced every 24 h, the effect was rescued at 48 h and 72 h, indicating that BECs are responsive to these signals and that these soluble factors may be labile or metabolized. However, the in vivo induction of P‐gp at the BBB by astrocytes is constant, as the release of many astrocyte‐secreted factors are dependent on neuronal activity (Otsu et al. 2015). Recently, astrocytes have been shown to be responsible for neurovascular coupling, a process by which blood flow is matched to neuronal activity (Devonshire et al. 2012). This coupling may regulate BBB properties through development. However, very little is known about how this process occurs in the developing brain or how it may affect BBB development.

It is clear that disruptions in astrocyte–neuron interaction contribute to the pathogenesis of neurodevelopmental disorders (Jacobs et al. 2010; Williams et al. 2014). However, this study highlights the importance of astrocyte–BBB interaction. Disruptions in astrocyte development may also dysregulate P‐gp at the developing BBB, which may contribute to the CNS dysfunction in neurodevelopmental disorders. Moreover, many conditions have been shown to alter the timing of astrocyte maturation. Prenatal chronic hypoxia and inflammation can delay the maturation of astrocytes in the fetal brain – an effect that has been shown to persist later in life (Tilleux and Hermans 2008; Raymond et al. 2011). Further investigation is needed to determine the effect of compromised astrocyte maturation on P‐gp function on the developing BBB.

There are a number of potential limitations to these types of in vitro study. The co‐culture model presented is a noncontact model, whereby BECs and astrocytes are not in physical contract and do not share a basement membrane. Moreover, the influence of other CNS cells, such as neurons and pericytes, are not taken into account. Previous studies have shown that these factors affect BEC and astrocyte function (Abbott 2002; Wolburg et al. 2009). Another limitation is the presence of serum in co‐culture media. Cells were grown in the presence of serum as BECs do not proliferate well in vitro without this component. However, in vivo astrocytes are not exposed to serum, future more focused studies will be designed to address these potential limitations. Notwithstanding, these studies do provide novel insight into the role of astrocytes in the regulation of P‐gp in the *developing* brain microvasculature.

In conclusion, astrocytes are key regulators of P‐gp expression and function at the developing BBB, but the magnitude of this effect is dependent on the developmental age of astrocytes. Aberrations in astrocyte maturation may alter levels of astrocyte‐derived factors and dysregulate P‐gp at the BBB. This may lead to altered movement of endogenous compounds, such as cytokines and steroid hormones, into the brain. Moreover, it is common for women to take prescribed drugs during pregnancy, many of which are P‐gp substrates. Altered levels of P‐gp at the developing BBB may result in increased fetal brain exposure to drugs and xenobiotics. Understanding how events during fetal life compromise astrocyte maturation and consequently P‐gp at the BBB will be critical in the development of future interventions to counteract these effects.

## Conflict of Interests

Authors do not have any professional or financial conflicts of interest.
